# A key role for *foxQ2* in anterior head and central brain patterning in insects

**DOI:** 10.1242/dev.147637

**Published:** 2017-08-15

**Authors:** Peter Kitzmann, Matthias Weißkopf, Magdalena Ines Schacht, Gregor Bucher

**Affiliations:** 1 Department of Evolutionary Developmental Genetics, GZMB, Universität Göttingen, Justus von Liebig Weg 11, 37077 Göttingen, Germany; 2 Department of Biology, Division of Developmental Biology, Friedrich-Alexander-University of Erlangen-Nürnberg, Staudtstraße 5, 91058 Erlangen, Germany

**Keywords:** *Tribolium castaneum*, Anterior gene regulatory network, Central complex development, FoxQ2, Head patterning, Six3

## Abstract

Anterior patterning of animals is based on a set of highly conserved transcription factors but the interactions within the protostome anterior gene regulatory network (aGRN) remain enigmatic. Here, we identify the red flour beetle *Tribolium castaneum* ortholog of *foxQ2* (*Tc-foxQ2*) as a novel upstream component of the aGRN. It is required for the development of the labrum and higher order brain structures, namely the central complex and the mushroom bodies. We reveal *Tc-foxQ2* interactions by RNAi and heat shock-mediated misexpression. Surprisingly, *Tc-foxQ2* and *Tc-six3* mutually activate each other, forming a novel regulatory module at the top of the aGRN. Comparisons of our results with those of sea urchins and cnidarians suggest that *foxQ2* has acquired more upstream functions in the aGRN during protostome evolution. Our findings expand the knowledge on *foxQ2* gene function to include essential roles in epidermal development and central brain patterning.

## Abstract (precis)

**Summary:** *foxQ2* and *six3* form a regulatory module at the top of the gene network governing anterior development of the *Tribolium castaneum* embryo.

## INTRODUCTION

Anterior patterning in bilaterian animals is based on a set of highly conserved transcription factors, such as *orthodenticle*/*otx*, *empty spiracles*/*emx*, *eyeless*/*Pax6* and other genes, which have comparable expression and function from flies to mice (Hirth et al., 1995; Leuzinger et al., 1998; Quiring et al., 1994; Simeone et al., 1992). Likewise, canonical Wnt signaling needs to be repressed in order to allow anterior pattern formation in most animals examined, with *Drosophila* being a notable exception (Fu et al., 2012; Martin and Kimelman, 2009; Petersen and Reddien, 2009). Recently, additional genes have been studied that are expressed anterior to the *orthodenticle/otx* region at the anterior pole of embryos of all major clades of Bilateria and their sister group, the Cnidaria. These data suggested that a distinct but highly conserved anterior gene regulatory network (aGRN) governs anteriormost patterning (Lowe et al., 2003; Marlow et al., 2014; Posnien et al., 2011b; Sinigaglia et al., 2013; Steinmetz et al., 2010; Yaguchi et al., 2008). This region gives rise to the apical organ in sea urchins, annelids and hemichordates, and other structures. Most of the respective orthologs were shown to be expressed in the vertebrate anterior neural plate as well as in the anterior insect head (Posnien et al., 2011b), although it remains disputed which, if any, tissue is homologous to the apical organ in these species (Hunnekuhl and Akam, 2014; Marlow et al., 2014; Santagata et al., 2012; Sinigaglia et al., 2013; Telford et al., 2008). Detailed interactions of this aGRN have been determined only in a few model systems (Range and Wei, 2016; Sinigaglia et al., 2013; Yaguchi et al., 2008).

The aGRN of sea urchins as representative of deuterostomes is best studied in *Strongylocentrotus purpuratus* and *Hemicentrotus pulcherrimus*. Here, *six3* (*sine oculis homeobox homolog 3/optix*) is the most upstream regulator, which is initially co-expressed with *foxQ2*. Both genes are restricted to the anterior pole by repression by posterior Wnt signaling (Range and Wei, 2016; Wei et al., 2009; Yaguchi et al., 2008). *six3* in turn is able to repress Wnt signaling (Wei et al., 2009) and to activate a large number of genes including *rx*, *nk2.1* and *foxQ2* (Wei et al., 2009). Subsequently, *foxQ2* represses *six3* but activates *nk2.1* expression at the anteriormost tip. In this tissue freed of *six3* expression, *foxQ2* is responsible for establishing a signaling center involved in the differentiation of the apical organ (Range and Wei, 2016). In addition, *foxQ2* expression is activated by nodal signaling (Yaguchi et al., 2016). *six3* knockdown leads to a strong morphological phenotype, including change of embryonic epidermal shape and loss of neural cells (Wei et al., 2009). In *foxQ2* knockdown, by contrast, an epidermal phenotype, other than animal plate thickening, was not observed (Yaguchi et al., 2008) but *foxQ2* appears to be essential for the specification of neural cell types (Yaguchi et al., 2008, 2012, 2016).

*Nematostella vectensis* (Cnidaria) represents the sister group to bilaterian animals (Sinigaglia et al., 2013). Here, both *Nv-six3* and *Nv-foxQ2* are initially activated by Wnt/β-catenin signaling, whereas at later stages there seems to be an antagonism between Wnt/β-catenin signaling and *Nv-six3* (Leclère et al., 2016). *Nv-six3* activates *Nv-foxQ2* and several other genes of the aGRN (Marlow et al., 2013; Sinigaglia et al., 2013). Like in the sea urchin, knockdown of *Nv-six3* leads to strong morphological defects including loss of the apical organ, whereas *Nv-foxQ2* knockdown does not affect the morphology of the embryo but the apical organ is reduced (Sinigaglia et al., 2013). In contrast to sea urchin, *Nv-foxQ2* does not appear to regulate *Nv-six3*. The repression of *six3* and *foxQ2* by Wnt signaling has been shown for a hemichordate (deuterostome) (Darras et al., 2011; Fritzenwanker et al., 2014) and for *foxQ2* in a hydrozoan (cnidarian) (Momose et al., 2008). Neither sea urchins nor cnidarians possess a highly centralized nervous system, such that a function of *foxQ2* in brain development could not be tested.

Within protostomes, the expression of aGRN genes has been studied extensively in postembryonic stages of the annelid *Platynereis dumerilii* (Lophotrochozoa). However, the only functional interaction tested was the repression of *Pd-six3* and *Pd-foxQ2* by Wnt signaling, which was activated by pharmacological treatment (Marlow et al., 2014). Within arthropods (Ecdysozoa), the red flour beetle *Tribolium castaneum* has become the main model system for studying anterior patterning (Posnien et al., 2010), because head development is more representative for insects than the involuted head of *Drosophila*. Indeed, the anterior morphogen *bicoid* is present only in some dipterans, while the canonical anterior repression of Wnt signaling is observed in *Tribolium* only (Brown et al., 2001; Stauber et al., 1999; Fu et al., 2012). Neither terminal Torso signaling nor the terminal gap gene *huckebein* has an influence on head formation in *Tribolium* (Schoppmeier and Schröder, 2005; Kittelmann et al., 2013). Apart from a region with similarity to vertebrate neural plate patterning there is also a largely non-neural anterior median region (AMR) patterned by other genes (Kittelmann et al., 2013; Posnien et al., 2011b). Interestingly, *Tc-six3* is a central regulator of anterior head and brain patterning, which represses *Tc-wg* expression, among other genes, but does not regulate *Tc-rx* or *Tc-nk2.1* (Posnien et al., 2011b).

In an ongoing genome-wide RNAi screen in *Tribolium* (iBeetle screen) (Dönitz et al., 2015; Schmitt-Engel et al., 2015) a head phenotype similar to that of *Tc-six3* RNAi was induced by the dsRNA fragment *iB_03837*. The targeted gene was the *Tribolium* ortholog of *foxQ2* (*Tc-foxQ2*), which encodes a Forkhead transcription factor. All members of this family share the Forkhead DNA-binding domain and they are involved in development and disease (Benayoun et al., 2011). Although highly conserved among animals, this gene was lost from placental mammals (Mazet et al., 2003; Yu et al., 2008). Within arthropods, anterior expression was described for the *Drosophila* ortholog *fd102C* (*CG11152*) (Lee and Frasch, 2004) and *Strigamia maritima* (myriapod) *foxQ2* (Hunnekuhl and Akam, 2014). However, the function of this gene has not been studied in any protostome so far.

We studied the expression and function of *Tc-foxQ2* by RNAi and heat shock-mediated misexpression and found that it has a much more central role in the insect aGRN than in sea urchin and cnidarians. Surprisingly, *Tc-foxQ2* and *Tc-six3* form a regulatory module with mutual activation, contrasting with the clear upstream role of *six3* in other species. Another difference is that *Tc-foxQ2* knockdown led to a strong epidermal phenotype. Further, we found a novel role of *Tc-foxQ2* in CNS patterning. Specifically, it was required for the development of the mushroom bodies (MBs) and the central complex (CX), both of which are higher-order processing centers of the insect brain (Heisenberg, 2003; Pfeiffer and Homberg, 2014). Finally, we present the most comprehensive aGRN available for protostomes.

## RESULTS

### *Tc-foxQ2* – a novel player in anterior head development of *Tribolium*

**Fig. 1.**
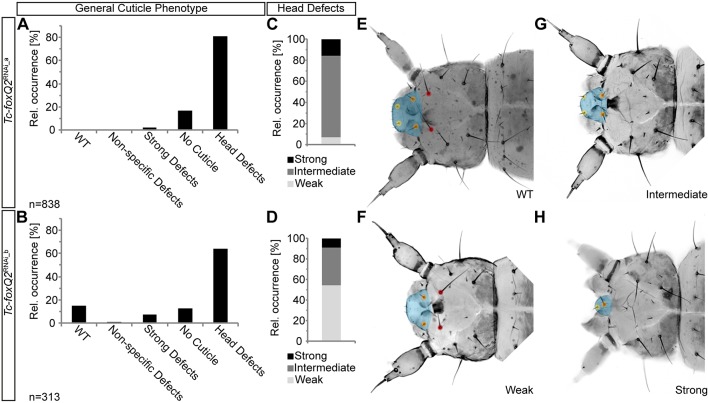
***Tc-foxQ2* RNAi phenotype and off-target control.** (A,B) Knockdown of *Tc-foxQ2* with the two non-overlapping dsRNA fragments *Tc-foxQ2*^RNAi_a^ (A) and *Tc-foxQ2*^RNAi_b^ (B) leads to comparable proportions of cuticle phenotypes. (C,D) Detailed analysis of the head defects shows that the *Tc-foxQ2*^RNAi_a^ dsRNA fragment leads to a qualitatively comparable but quantitatively stronger phenotype, marked by more intermediate and strong head defects. (E-H) L1 cuticle heads representing the different classes of head defect. Dorsal view, anterior left. (E) WT cuticle with the labrum marked in cyan, two labrum setae (yellow dots), two clypeus setae (orange dots) and two anterior vertex setae (red dots). (F) Weak head defect with a reduced labrum and at least one deleted labrum seta. (G) Intermediate head defect, additionally lacking at least one of the anterior vertex seta. (H) Strong head defect with a strongly reduced labrum, one labrum seta and one clypeus seta. In the strongest phenotypes the labrum and the anterior vertex setae are deleted. Non-specific defects refers to local alterations in cuticular structures that are most likely not due to the RNAi treatment. Strong defects refers to severe alterations affecting all tagma that are most likely not due to the RNAi treatment.

In the iBeetle screen, injection of the dsRNA fragment *iB_03837* led to first instar larval cuticles with reduced or absent labrum (cyan area in Fig. 1) with high penetrance (Dönitz et al., 2015; Schmitt-Engel et al., 2015) (Fig. 1A). The targeted gene was *TC004761* (Tcas_OGS 3.0), which was revealed as the sole *Tribolium* ortholog of *foxQ2* (*Tc-foxQ2*) (Fig. S1).

Quantitative analyses of parental RNAi experiments with two non-overlapping dsRNA fragments (*Tc-foxQ2*^RNAi_a^ and *Tc-foxQ2*^RNAi_b^; 1.5 µg/µl) (Fig. 1A,B, Tables S1-S4) in two different genetic backgrounds (Fig. S2, Tables S3-S6) revealed the same morphological phenotype, arguing against off-target effects or strong influence of the genetic background (Kitzmann et al., 2013). The proportions of eggs without cuticles or with cuticle remnants (strong defects) were within the range observed in wild type (WT) (van der Zee et al., 2005). Weak *Tc-foxQ2* RNAi phenotypes (Fig. 1C,D) showed a labrum that was reduced in size and loss of one or both labral setae (Fig. 1E,F, yellow dots). Intermediate phenotypes (Fig. 1C,D) were marked by a reduced labrum and loss of one or both anterior vertex triplet setae (Fig. 1E,G, red dots), indicating additional deletions of the head capsule. In strong phenotypes (Fig. 1C,D), the labrum was substantially reduced or deleted along with several setae marking the anterior head and/or the labrum (Fig. 1E,H). No other specific L1 cuticle phenotypes were detected. We tested higher dsRNA concentrations (2 µg/µl and 3.1 µg/µl; data not shown) as well as double RNAi using both dsRNA fragments together (1.5 µg/µl each; data not shown). None of these variations resulted in a stronger cuticle phenotype. Taken together, *Tc-foxQ2* is required for epidermal patterning of anterior head structures. Interestingly, the RNAi phenotype was similar to that of *Tc-six3*, although somewhat weaker and less penetrant (Posnien et al., 2011b).

### Increased apoptosis in *Tc-foxQ2* RNAi

**Fig. 2.**
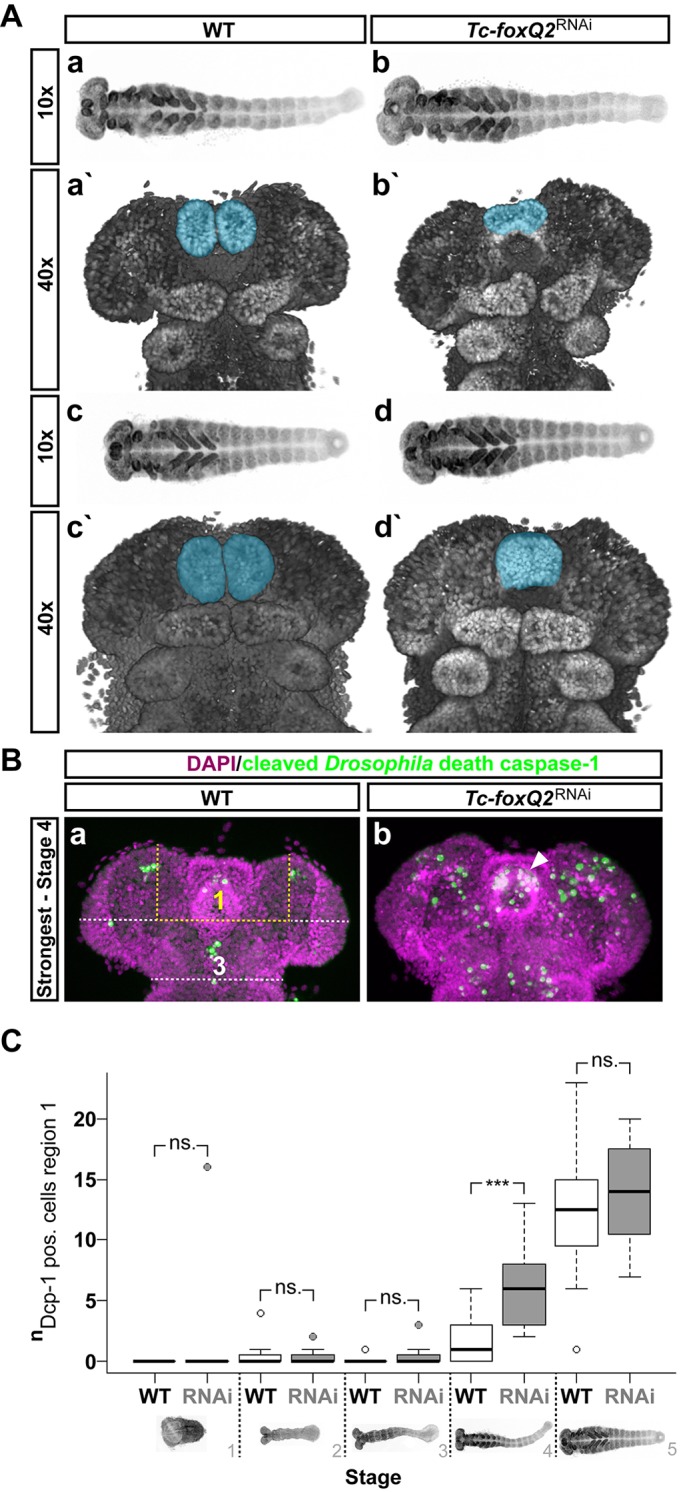
**Cell death in the *Tc-foxQ2*^RNAi^ phenotype.** (A) Morphology of WT (Aa,a′,c,c′) and *Tc-foxQ2*^RNAi^ (Ab,b′,d,d′) embryos is visualized by nuclear staining (DAPI, gray). Anterior is left in 10× and up in 40× panels. Aa,b represent late elongating germ bands; Ac,d show early retracting germ bands. The labrum is marked in blue. *Tc-foxQ2*^RNAi^ embryos (6-26 h AEL) show decreased labral buds, which appear to fuse prematurely (Ab′,d′). (B) For quantification of cell death, a region of interest (region 1) and a control region (region 3) were defined. Apoptotic cells were monitored by Dcp-1 antibody staining (green). A fully elongated *Tc-foxQ2*^RNAi^ WT germ band with most apoptotic cells within the labral region (Ba, region 1) is compared with a *Tc-foxQ2*^RNAi^ embryo that shows most apoptotic cells within region 1 (Bb, arrowhead). (C) Box plot depicting the normalized number of apoptotic cells at five different embryonic stages for untreated (WT) and *Tc-foxQ2*^RNAi^ embryos (RNAi). The values are normalized by region 3 values. Germ rudiments (stage 1) to intermediate elongating germ bands (stage 3), as well as early retracting germ bands (stage 5), show no significant increase in the number of apoptotic cells. Stage 1, *P*=0.33 (WT *n*=3, RNAi *n*=7); stage 2, *P*=0.63 (WT *n*=11, RNAi *n*=12); stage 3, *P*=0.19 (WT *n*=9, RNAi *n*=19); stage 5, *P*=0.15 (WT *n*=12, RNAi *n*=11). However, fully elongated germ bands showed significantly more apoptotic cells (***P=4.1×10^−4^) in *Tc-foxQ2*^RNAi^ embryos (*n*=15) than in untreated embryos (*n*=17). The line inside the box represents the median, the box is defined by the first and the third quartiles, the whiskers are defined as within 1.5× the interquartile range of the lower/upper quartile and dots represent outliers. ns., not significant.

The labrum is an appendage-like structure (Posnien et al., 2009a) and its outgrowth requires cell proliferation regulated by *Tc-serrate* (*Tc-ser*) (Siemanowski et al., 2015). We examined when the size of the labrum decreased after *Tc-foxQ2* RNAi and whether cell proliferation or cell death was involved. We found that the labral buds in *Tc-foxQ2*^RNAi^ embryos were decreased and fused from fully elongated germ band stages onwards (Fig. 2Aa′-d′). We found no regulation of *Tc-ser* by *Tc-foxQ2* (see below).

Next, we quantified apoptosis in embryos 6-26 h after egg laying (AEL) (Table S7) using an antibody against cleaved death caspase-1 (Dcp-1) (Florentin and Arama, 2012). We quantified apoptotic cells in the labral region (region 1 in Fig. 2B) and a control region (region 3). Fully elongated germ bands showed a 6-fold increase in the number of apoptotic cells in the labral region compared with the control region after *Tc-foxQ2* RNAi (*P=*4.1×10^−4^, *n*=15; Fig. 2C) coinciding with the stage of morphological reduction. Hence, *Tc-foxQ2* prevents apoptosis in the growing labrum.

### *Tc-foxQ2* is required for brain development

**Fig. 3.**
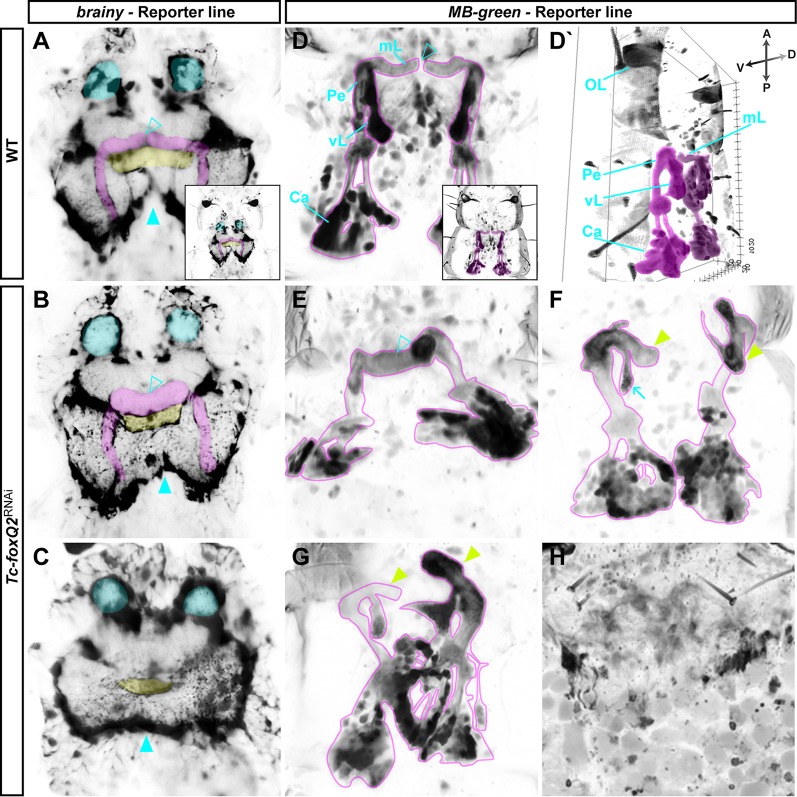
**Loss of *Tc-foxQ2* function leads to brain defects.** (A-C) Glial tissue (black) is visualized in L1 larvae by the transgenic *brainy* reporter line in WT (A) and *Tc-foxQ2*^RNAi^ (B,C). Voltex projections. (D-H) Mushroom bodies (MBs; black) are visualized by the transgenic *MB-green* reporter line in WT (D,D′) and *Tc-foxQ2*^RNAi^ (E-H). MAX projections; D′, 3D projection. (A) WT L1 larval brain showing the two brain hemispheres with MBs (magenta), antennal lobes (cyan), and the midline-spanning central body (CB; yellow). (B) A weak *Tc-foxQ2*^RNAi^ larval brain phenotype showing loss of the boundary between the medial lobes of the MB (compare open arrowheads in B and A). The CB appears to be reduced in size. (C) Intermediate phenotype *Tc-foxQ2*^RNAi^ larval brains appear to lack the MBs and the CB appears reduced in size. (B,C) In both phenotypes the brain hemispheres appear to be fused (solid arrowhead). (D) WT L1 larval MBs in dorsal view. (D′) 3D projection of WT L1 larval MBs, providing an overview of the organization of the structures. (E) A weak *Tc-foxQ2* RNAi-induced MB phenotype, where the two medial lobes appear to be fused (compare arrowheads in D and E). (F) MBs with distorted pedunculi, leading to a loss of contact between the two medial lobes (arrowheads), and reduced vertical lobes (arrow). (G) Phenotype with interdigitating MBs. (H) In strong phenotypes the MB structures are highly reduced or absent. OL, optical lobe; mL, medial lobe; Pe, pedunculus; vL, vertical lobe; Ca, calyx.

The phenotypic similarity to *Tc-six3*^RNAi^ embryos (Posnien et al., 2011b) and the expression of *Tc-foxQ2* in neuroectodermal tissue (see below) prompted us to check for brain phenotypes. We performed parental RNAi in the background of the *brainy* line, which marks glia by ECFP (black signal in Fig. 3A-C) (Koniszewski et al., 2016). In the weakest phenotypes, the medial lobes of the MBs were reduced and appeared to be medially fused (Fig. 3, compare open arrowheads in A with B). Further, the central body (CB; part of the CX) was shortened (Fig. 3B) and the brain hemispheres were slightly fused (Fig. 3, compare solid arrowheads in B with A). In stronger phenotypes, the CB was clearly reduced in size and the MBs were not detectable. Further, the brain hemispheres appeared fused at the midline (Fig. 3C).

We performed RNAi in the background of the transgenic *MB-green* line, which marks MBs by EGFP (Koniszewski et al., 2016) (the magenta-framed black signal in Fig. 3D-H). We observed a similar range of MB body phenotypes. In addition to fused MBs, we found misarranged MBs that had lost their medial contact (Fig. 3F,G, arrowheads), interdigitated MBs (Fig. 3G), and highly reduced or even absent MBs (Fig. 3H). Importantly, the strength of epidermal and neural phenotypes correlated. Larvae with weak neural defects showed a decreased labrum, while strong neural phenotypes correlated with lack of the entire labrum. Taken together, we found *Tc-foxQ2* to be required for brain formation, with the MBs, CB and the midline being strongly affected. Again, these defects are similar to those reported for *Tc-six3* loss of function (Posnien et al., 2011b).

### Dynamic expression of *Tc-foxQ2* in the anterior head

**Fig. 4.**
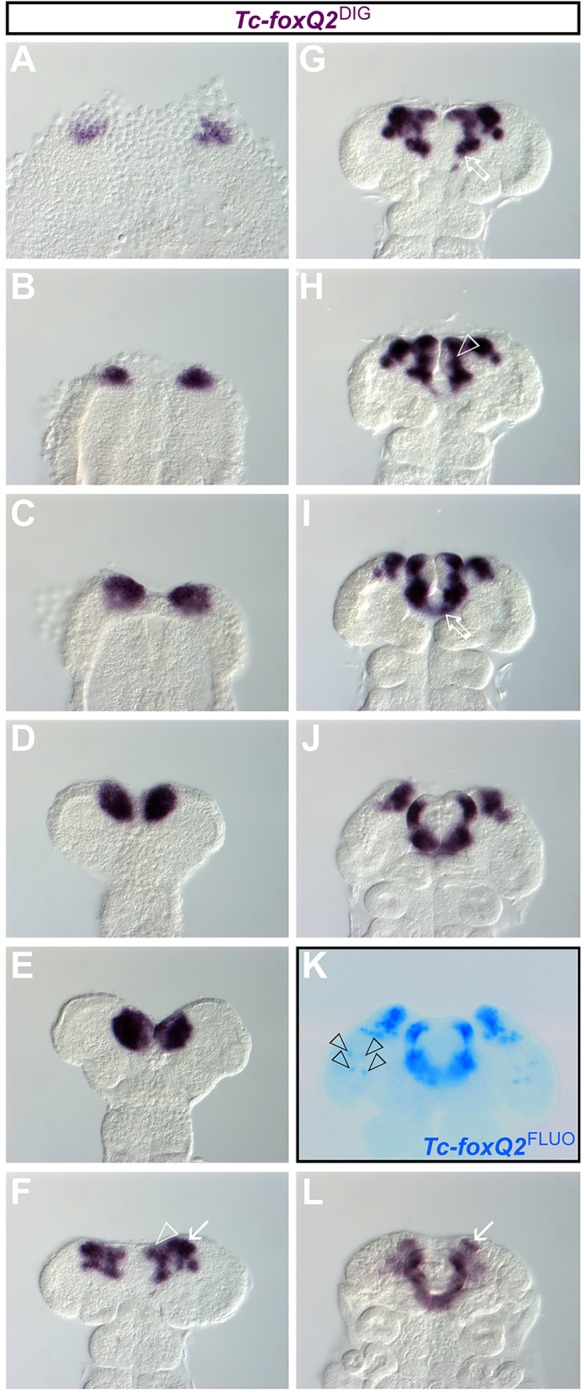
***Tc-foxQ2* is expressed in a highly dynamic pattern at the anterior pole.** Expression of *Tc-foxQ2* in WT embryos monitored by whole-mount *in situ* hybridization (ISH). Anterior is up in all images. (A) *Tc-foxQ2* expression starts with the formation of the germ rudiment. (A-E) The early *Tc-foxQ2* expression starts with two domains located at the anterior pole, which successively approach each other, probably as a consequence of morphogenetic movements. (F) The expression splits into several domains in late elongating germ bands, with expression in the putative neuroectoderm (arrow, presumably including parts of the pars intercerebralis; Posnien et al., 2011b) and in the labral/stomodeal region (arrowhead). (G) The expression domains flanking the prospective stomodeum become more prominent (arrow). (H) The anterior median expression domain frames the lateral parts of the labral buds (arrowhead). (I,J) At fully elongated and early retracting germ band stages the two expression domains flanking the stomodeum become posteriorly linked (arrow). (K) Staining with the more sensitive TSA-Dylight550 reveals four dot-like expression domains in the ocular region (arrowheads). (L) At retracting germ band stages *Tc-foxQ2* is expressed in a narrow U-shaped pattern and the neuroectodermal expression domains are reduced in size (arrow).

The expression of *Tc-foxQ2* started in two domains at the anterior terminus of the germ rudiment (Fig. 4A,B). During elongation, the two domains approach each other as a consequence of morphological movements (Fig. 4C-E). At late elongating germ band stages, the domains split into several subdomains in the AMR and the labrum anlagen (arrowhead in Fig. 4F,H) and domains lateral to the stomodeum (arrow in Fig. 4G,I,J); in addition, there are domains in the neuroectoderm (e.g. arrow in Fig. 4F,L; see approximate fate map in Fig. S3). Very weak staining in the ocular region was detected using the tyramide signal amplification (TSA) system (Fig. 4K). These data are comparable to the anterior expression of *foxQ2* orthologs in other animals and are consistent with the phenotype in *Tribolium*.

**Fig. 5.**
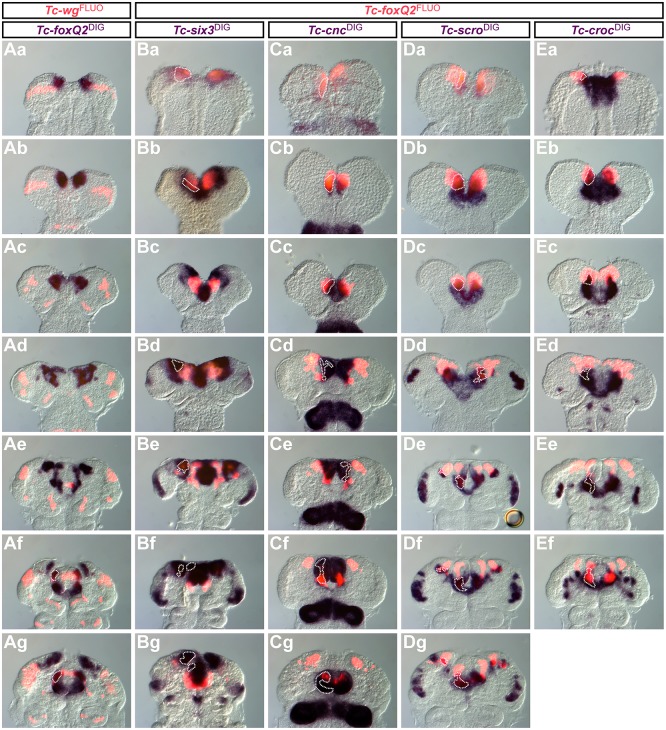
**Co-expression of *Tc-foxQ2* and anterior** **head patterning genes.** Expression is visualized by double ISH, using NBT/BCIP (blue) and TSA-Dylight550 (red). Regions of co-expression are outlined. Anterior is up in all images. (Aa-g) Co-expression of *Tc-foxQ2* with *Tc-wg* is found only after full elongation (Af,g). (Ba-g) *Tc-foxQ2* and *Tc-six3* expression completely overlap during germ rudiment stages (Ba). In early elongating germ bands the co-expression is limited to a narrow lateral stripe of the anterior median region (AMR) (Bb). Intermediate germ bands show mutually exclusive expression of *Tc-foxQ2* and *Tc-six3* (Bc). At later stages, expression overlap is found within the neurogenic region (Bd,e) and later also in the labral buds (Bf,g). (Ca-g) *Tc-cnc* expression is completely overlapping with *Tc-foxQ2* at early embryonic stages (Ca-c) but later co-expression is found in the labral/stomodeal region (Cd-g). (Da-g) *Tc-scro* is partially co-expressed at early embryonic stages (Da-c). In late elongating germ bands the co-expression is restricted to a narrow lateral stripe (Dd) and the posterior portion of the labral buds, close to the stomodeum and small areas of the neurogenic region (De-g). (Ea-g) *Tc-croc* expression is partially overlapping with *Tc-foxQ2* at early stages (Ea-c) and later in a region close to the stomodeum (Ed-f).

We mapped the expression of *Tc-foxQ2* relative to other genes of the aGRN by double *in situ* hybridization in WT embryos. Expression overlaps are outlined (dotted lines) in Fig. 5 and Fig. S4. First, we tested for genes that interact with *foxQ2* in other species or are required for labrum formation (Coulcher and Telford, 2012; Economou and Telford, 2009; Kittelmann et al., 2013; Posnien et al., 2009a,b). We found no overlap with *Tc-wingless/wnt1* (*Tc-wg*) expression until retraction, when the emerging stomodeal *Tc-wg* domain overlapped with *Tc-foxQ2* expression (Fig. 5A). By contrast, we found complete overlap with *Tc-six3* expression at early embryonic stages (Fig. 5Ba), which developed into a mutually exclusive expression at intermediate elongating germ bands (Fig. 5Bc). Thereafter, these genes remained mutually exclusive, except for a small anterior median neuroectodermal region (lateral area marked in Fig. 5Bd-g) and the labrum anlagen (median area marked in Fig. 5Bf,g). These data are in agreement with the interactions described for sea urchin, where *six3* initially activates *foxQ2* but at later stages *six3* becomes repressed by *foxQ2* (see Introduction). The later coexistence of mutually exclusive and co-expression domains along with the many different expression domains of *Tc-foxQ2* indicate a complex and region-specific regulation. Early co-expression developing into partially overlapping expression patterns was observed for both *Tc-cap‘n'collar* (*Tc-cnc*) and *Tc-scarecrow/nk2.1* (*Tc-scro*) (Fig. 5C,D). For *Tc-crocodile* (*Tc-croc*), a small overlap was observed, which remained throughout development (Fig. 5E).

Next we studied genes that, based on their expression and function, are thought to be downstream components of the aGRN. *Tc-retinal homeobox* (*Tc-rx*) was expressed in a largely non-overlapping pattern, apart from small domains in the labrum and neuroectoderm at late stages (Fig. S4A). Expression of *Tc-chx* and *Tc-ser* initially largely overlaps with that of *Tc-foxQ2*, but they later resolve to mainly non-overlapping patterns (Fig. S4B,E). *Tc-forkhead/foxA* (*Tc-fkh*) expression was essentially non-overlapping with *Tc-foxQ2* (Fig. S4C). *Tc-six4* marks a region with molecular similarity to the vertebrate placodes (Posnien et al., 2011a). No co-expression was observed until late stages, when a small domain expresses both genes (Fig. S4D). In summary, *Tc-foxQ2* expression appears to have a central and dynamic role in the aGRN.

### *Tc-foxQ2* is required for *Tc-six3* expression and is repressed by Wnt signaling

**Fig. 6.**
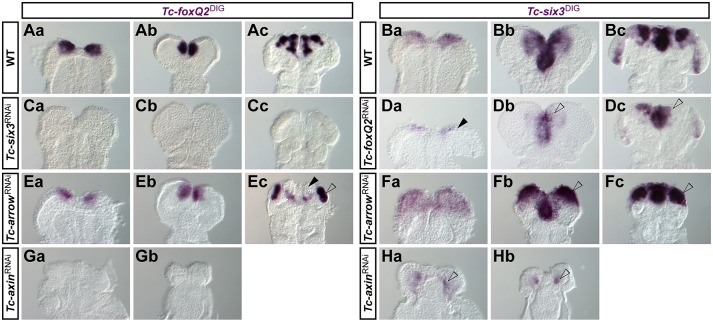
**Mutual activation of *Tc-foxQ2* and *Tc-six3* and their repression by Wnt signaling.** Expression pattern of *Tc-foxQ2* in WT (Aa-c), *Tc-six3*^RNAi^ (Ca-c), *Tc-arr*^RNAi^ (Ea-c) and *Tc-axin*^RNAi^ (Gb,c) embryos and expression pattern of *Tc-six3* in WT (Ba-c), *Tc-foxQ2*^RNAi^ (Da-c), *Tc-arr*^RNAi^ (Fa-c), and *Tc-axin*^RNAi^ (Ha,b) embryos monitored by ISH. (Ca-c) *Tc-foxQ2* expression was lost in *Tc-six3*^RNAi^ embryos. (Da) *Tc-six3* expression was strongly reduced in *Tc-foxQ2*^RNAi^ germ rudiments (arrowhead). (Db,c) Later, median expression emerged but the lateral *Tc-six3* expression remained reduced or absent (arrowhead), whereas the ocular domain appeared unchanged. (Ea,b) *Tc-foxQ2* expression was unchanged in early *Tc-arr*^RNAi^ embryos but the median *Tc-foxQ2* expression was reduced at later stages (Ec, solid arrowhead), whereas the neurogenic *Tc-foxQ2* expression domain expanded (Ec, open arrowhead). (Fa-c) In *Tc-arr*^RNAi^ embryos the *Tc-six3* expression expanded towards the posterior in the neuroectoderm (arrowheads), whereas the stomodeal domain appeared to be unaffected. In *Tc-axin*^RNAi^ embryos Wnt signaling is derepressed, which led to repression of *Tc-foxQ2* expression (Ga,b) and to a strong reduction of *Tc-six3* expression (Ha,b, arrowheads).

We first tested the interactions of *foxQ2* known from other species. *Tc-foxQ2* was virtually absent in *Tc-six3* RNAi embryos (Fig. 6 Ca-c, compare with Aa-c) indicating a conserved role of *Tc-six3* in *Tc-foxQ2* activation. Only at later stages was some residual *Tc-foxQ2* expression seen in the stomodeal region (not shown). Unexpectedly, *Tc-six3* expression was strongly reduced in *Tc-foxQ2*^RNAi^ germ rudiments (compare Fig. 6Ba with Da), which contrasts with findings in cnidarians and sea urchins. At later stages, medial *Tc-six3* expression emerged (Fig. 6Db) and developed a similar shape and intensity as in WT (Fig. 6Dc). However, the lateral neuroectodermal domains remained strongly reduced or even absent (Fig. 6Db,c, arrowheads). Efficient knockdown of *Tc-foxQ2* by RNAi was shown by *in situ* hybridization and RT-qPCR (Figs S5 and S6). The reduction of *Tc-six3* in *Tc-foxQ2*^RNAi^ animals was confirmed by RT-qPCR experiments (–25.6±0.05%; Fig. S6).

At early embryonic stages, *Tc-wg* expression was not altered in *Tc-foxQ2*^RNAi^ (not shown), whereas later the labral *Tc-wg* expression was lost (Fig. S7). Likewise, inhibition of canonical Wnt signaling by *Tc-arr* RNAi (Bolognesi et al., 2009) did not alter *Tc-foxQ2* expression at early stages (Fig. 6Ea,b). Later, the neuroectodermal *Tc-foxQ2* expression domains were expanded (Fig. 6Ec, open arrowhead), whereas the anterior labral *Tc-foxQ2* expression domain was lost (Fig. 6Ec, solid arrowhead). The *Tc-six3* expression domains were expanded in *Tc-arr*^RNAi^ germ rudiments covering the anterior region (Fig. 6Fa). Later, the neuroectodermal *Tc-six3* expression domains were strongly expanded (Fig. 6Fb,c, arrowheads), whereas the median domains were unchanged. Overactivation of canonical Wnt signaling via *Tc-axin* RNAi led to a strong reduction of *Tc-foxQ2* (Fig. 6Ga,b) and *Tc-six3* (Fig. 6Ha,b) expression. In summary, as in other model systems, we found activation of *Tc-foxQ2* by *Tc-six3* and repression by Wnt signaling. In contrast to other clades, *Tc-foxQ2* was required for early *Tc-six3* expression.

### *Tc-foxQ2* acts upstream in anterior AMR patterning

**Fig. 7.**
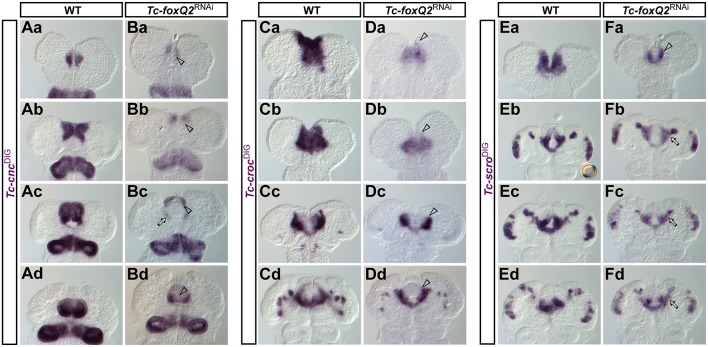
***Tc-foxQ2*^RNAi^ regulates *Tc-cnc*, *Tc-croc* and *Tc-scro* expression.** Expression pattern of *Tc-cnc* in WT (Aa-d) and *Tc-foxQ2*^RNAi^ (Ba-d) embryos, expression of *Tc-croc* in WT (Ca-d) and *Tc-foxQ2*^RNAi^ (Da-d) embryos, and expression of *Tc-scro* in WT (Ea-d) and *Tc-foxQ2*^RNAi^ (Fa-d) embryos monitored by ISH. (Ba,b) In *Tc-foxQ2*^RNAi^ embryos the anterior domain of *Tc-cnc* expression was reduced (arrowheads). Prior to this stage, no changes in the expression pattern were observed (not shown). (Bc,d) In fully elongated and retracting germ bands the labral expression was strongly reduced (arrowheads), while stomodeal expression was slightly altered (arrow). (Da-d) Throughout development, the *Tc-croc* expression pattern was lacking the anterior portion of its AMR expression domain (arrowheads). (Fa) Expression of *Tc-scro* is reduced to a narrow stripe along the anterior fold (arrowhead). (Fb-d) Later stages show an atypical bridging between the labral/stomodeal and the neurogenic *Tc-scro* expression domains (arrows).

The AMR of the insect head harbors the labrum and the stomodeum. *Tc-cnc* and *Tc-croc* are upstream factors required for anterior and posterior AMR patterning, respectively (Economou and Telford, 2009; Hunnekuhl and Akam, 2014; Kittelmann et al., 2013). The AMR expression domain of *Tc-cnc* in the labrum was strongly reduced after knockdown of *Tc-foxQ2* (Fig. 7Ba-d, compare with Aa-c). Likewise, *Tc-croc* expression was affected, but the reduction was restricted to the anterior boundary of expression (arrowheads in Fig. 7Da-d, compare with Ca-c); posterior expression around the stomodeum was largely unchanged. Conversely, in *Tc-croc* and *Tc-cnc* RNAi we observed no alteration of *Tc-foxQ2* expression at early stages (not shown), indicating an upstream role of *Tc-foxQ2*. However, at later stages, expression of *Tc-foxQ2* was reduced in the labrum in both treatments (Fig. S8). Next, we tested the medial AMR markers *Tc-scro* and *Tc-fkh*. *Tc-scro* was reduced anteriorly and laterally in *Tc-foxQ2*^RNAi^ embryos in early elongating germ bands (Fig. 7Fa, arrowhead, compare with Ea), but its posterior aspects were only moderately altered. In contrast to WT embryos, in *Tc-foxQ2*^RNAi^ the stomodeal/labral expression of *Tc-scro* remained connected to the lateral expression in neuroectoderm (Fig. 7Fb-d, arrows, compare with Eb-d). Conversely, *Tc-foxQ2* was not altered in early *Tc-scro*^RNAi^ embryos, whereas in later embryos changes were observed (Fig. S8). The expression of the stomodeum marker *Tc-fkh* was not considerably altered in *Tc-foxQ2*^RNAi^ embryos (Fig. S9), whereas later aspects of *Tc-foxQ2* expression were altered upon *Tc-fkh* RNAi, probably by indirect effects (Fig. S9).

The Notch pathway ligand *Tc-ser* and the ubiquitin ligase *Tc-mind bomb1* (*Tc-mib1*) are required for Notch signaling, and knockdown of both leads to loss of the labrum (Siemanowski et al., 2015). However, we detected no difference in *Tc-ser* expression in *Tc-foxQ2*^RNAi^ (not shown). Conversely, in early and intermediate elongating *Tc-mib1*^RNAi^ embryos only the lateral aspects of *Tc-foxQ2* expression appeared mildly decreased (Fig. S10), arguing against a strong interaction with the Notch pathway. At later stages, by contrast, some lateral and labral *Tc-foxQ2* expression domains were clearly reduced in *Tc-mib1*^RNAi^ embryos. Taken together, these results demonstrated an upstream role of *Tc-foxQ2* in early anterior AMR patterning and indicated that the later interactions of the aGRN differ from the early interactions.

### An upstream role of *foxQ2* in neuroectoderm patterning

**Fig. 8.**
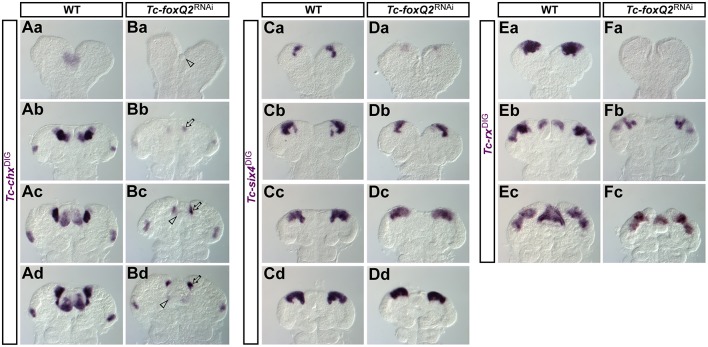
***Tc-foxQ2*^RNAi^ embryos show reduced *Tc-chx*, *Tc-six4* and *Tc-rx* expression.** Expression patterns of *Tc-chx* in WT (Aa-d) and *Tc-foxQ2*^RNAi^ embryos (Ba-d), of *Tc-six4* in WT (Ca-d) and *Tc-foxQ2*^RNAi^ embryos (Da-d) and of *Tc-rx* in WT (Ea-c) and *Tc-foxQ2*^RNAi^ embryos (Fa-c) as monitored by ISH. (Ba) *Tc-chx* expression was completely absent in early elongating *Tc-foxQ2*^RNAi^ germ bands (arrowhead). (Bb-d) At later stages, the labral *Tc-chx* expression domains were almost absent (arrowheads), while the anterior neurogenic expression domains were strongly reduced (arrows). The ocular *Tc-chx* expression domain remained unaffected. (Da) Expression of *Tc-six4* is strongly reduced in early elongating germ bands (arrow). (Db-d) At later stages, only the median posterior extensions of the *Tc-six4* expression domains were reduced (arrows). (Fa) *Tc-rx* expression is strongly reduced or completely absent in early elongating *Tc-foxQ2*^RNAi^ germ bands. (Fb,c) At later stages, the neurogenic *Tc-rx* expression pattern appeared unaffected, but the labral expression domains were absent (Fb, arrowhead) or reduced in size (Fc, arrowhead). Anterior is up in all images.

The anterior neuroectoderm is marked by the expression of several highly conserved transcription factors and harbors the anlagen of the insect head placode (Posnien et al., 2011a), the pars intercerebralis and the pars lateralis (Posnien et al., 2011b) (Fig. S3). Furthermore, it corresponds to the region where in grasshoppers several neuroblasts arise, which are required for CX development (Boyan and Reichert, 2011). *Tc-chx* expression was completely lost in early elongating *Tc-foxQ2*^RNAi^ germ bands (Fig. 8Ba, arrowhead, compare with Aa) and highly reduced at later stages (arrows in Fig. 8Bb-d, compare with Ab-d). The *Tc-six4* domain was much reduced in early *Tc-foxQ2*^RNAi^ embryos, showing only small spots of expression at the anterior rim (Fig. 8Da, arrow, compare with Ca). Later, the lateral expression developed normally, whereas a median aspect of its expression was lost (Fig. 8Db-d, arrows, compare with Cb-d). *Tc-rx* expression at early elongating germ band stages was absent after *Tc-foxQ2*^RNAi^ (Fig. 8Fa, compare with Ea). This was unexpected because *Tc-rx* is largely expressed outside the *Tc-foxQ2* expression domain (Fig. S4Aa-c), arguing against a direct effect. Indeed, our misexpression studies indicate a repressive role (see below). At later stages, the lateral aspects of *Tc-rx* expression recovered but the labral expression domain remained reduced or lost, in line with labral co-expression (Fig. 8Fb,c, arrowheads, compare with Eb,c).

Next, we scored *Tc-foxQ2* expression in *Tc-chx*^RNAi^, *Tc-six4*^RNAi^ and *Tc-rx*^RNAi^ embryos. In neither treatment were early aspects of *Tc-foxQ2* expression affected, whereas at later stages we found expression differences within the neurogenic region (Fig. S11). These results confirm the upstream role of *Tc-foxQ2* in early anterior patterning and they confirm that the interactions of the aGRN at later stages differ from those at early stages. We found no change in cell death in the neurogenic region of *Tc-foxQ2*^RNAi^ embryos until retraction, when cell death was significantly increased 1.5-fold (*P*=0.023) (Fig. S12). Hence, the observed changes in expression domains are likely to be due to regulatory interactions and not loss of tissue.

### *Tc-foxQ2* gain-of-function analysis confirms a function in anterior median neuroectoderm

Heat shock-mediated misexpression has been established in *Tribolium* (Schinko et al., 2012). We generated eight independent transgenic lines and selected one for our experiments that showed the most evenly distributed misexpression upon heat shock (Fig. S13). Heat shock-induced misexpression led to a reproducible pleiotropic cuticle phenotype. The bristle pattern of the anterior head showed diverse signs of mild disruption (Fig. S14). Ectopic *Tc-foxQ2* expression led to a reduced number of segments in the legs, the abdomen and the terminus. For our experiments, we used the earliest possible time point of misexpression (9-13 h AEL), which led to a higher incidence of anterior defects than at 14-20 h AEL and 20-25 h AEL (not shown). At 14-18 h AEL (5 h after a 10 min heat shock) the embryos were fixed and marker gene expression was scored. WT embryos undergoing the same procedure were used as negative control.

Heat shock-induced expression starts at late blastoderm stages (Schinko et al., 2012). Therefore, we were not able to test for the early interactions of the aGRN. Hence, comparably mild alterations in expression were found for *Tc-wg*, *Tc-six3* and *Tc-croc* after ectopic *Tc-foxQ2* expression (Fig. S15).

**Fig. 9.**
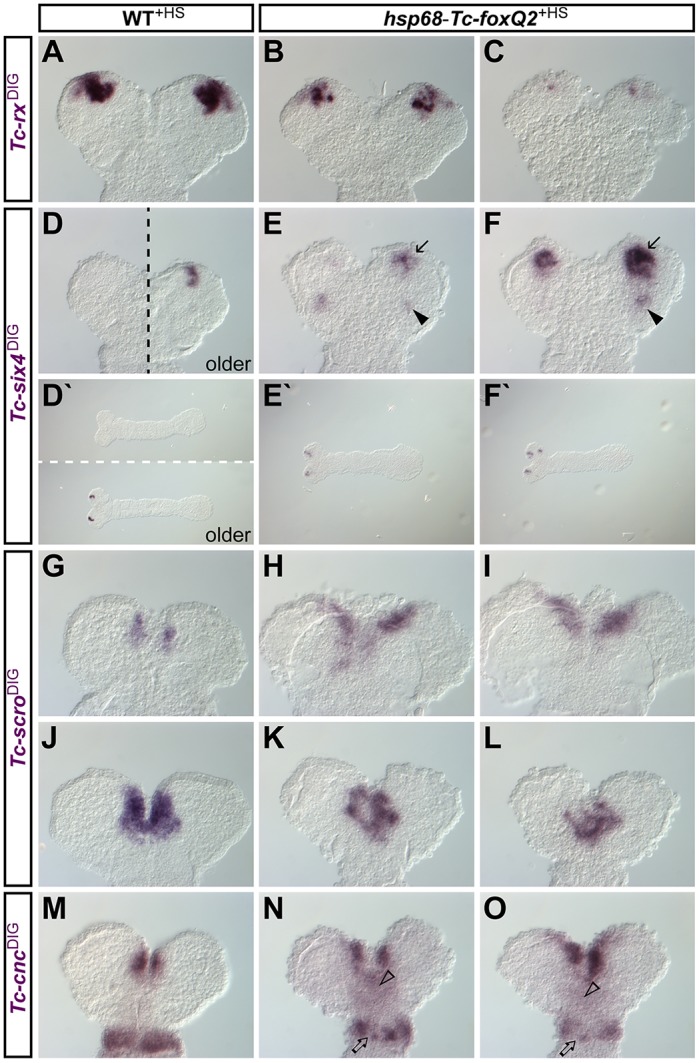
**Ectopic *Tc-foxQ2* expression impacts head patterning.** Expression of head patterning genes in heat shock-treated WT (A,D,D′,G,J,M) and *hsp68*-*Tc-foxQ2* (B,C,E,E′,F,F′,H,I,K,L,N,O) embryos (14-18 h AEL) as monitored by ISH. (B,C) Ectopic *Tc-foxQ2* expression led to reduced *Tc-rx* expression. (E,F) *Tc-six4* expression showed a premature onset at the anterior tip (arrows; compare embryonic stages of E′,F′ with D′). Further, this premature expression was expanded; and an additional, more posterior *Tc-six4* expression domain emerged (arrowheads). (H,I) *Tc-scro* expression started prematurely and was expanded. (K,L) By contrast, early elongating germ bands showed reduced *Tc-scro* expression – presumably a secondary effect. (N,O) The anterior *Tc-cnc* expression domain spread towards the posterior (arrowheads). The mandibular *Tc-cnc* expression domain was reduced and became somewhat punctate (arrows). Anterior is up in all panels except D′-F′**,** where anterior is to the left.

The strongest effects were on genes with late-onset expression, i.e. *Tc-rx*, *Tc-six4*, *Tc-scro* and *Tc-cnc* (Fig. 9). *Tc-rx* expression was reduced to a punctate pattern (Fig. 9B,C, compare with A). This repressive function of *Tc-foxQ2* on *Tc-rx* is in line with the non-overlapping, adjacent expression of these two genes (Fig. S4Aa-c). Therefore, we assume that the reduction of *Tc-rx* found in *Tc-foxQ2* RNAi is due to secondary effects, although this remains speculative (see above). Ectopic *Tc-foxQ2* expression caused a premature onset and an expansion of *Tc-six4* expression (Fig. 9D,F) and an additional ectopic domain was found in the posterior head (Fig. 9E,F, arrowhead). *Tc-scro* expression emerged precociously and was expanded (Fig. 9H,I and K,L; compare with G and J, respectively). Later, the domain was altered in shape and had a punctate appearance. In the case of *Tc-cnc*, a posterior expansion in expression was observed (Fig. 9N,O, arrowhead, compare with M).

## DISCUSSION

### Dynamic *six3*/*foxQ2* interactions govern anterior development

**Fig. 10.**
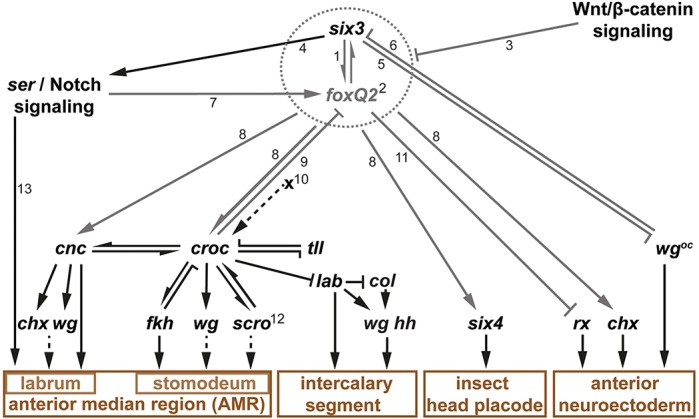
***Tc-foxQ2* and *Tc-six3* form a regulatory module in the aGRN.** Black lines indicate previously reported interactions (based on: Kittelmann et al., 2013; Posnien et al., 2011b; Schaeper et al., 2010; Siemanowski et al., 2015). Arrows represent gene activation, and cross-bars indicate gene repression. This aGRN represents the interactions at early embryonic stages – later interactions are likely to differ. (1) *Tc-six3* is the most upstream factor for patterning the anterior median head and neuroectoderm. (2) *Tc-foxQ2*, like *Tc-six3*, is a key player in anterior head development, with a somewhat later onset of expression than *Tc-six3*. Mutual activation, similar effects on other genes and similar phenotypes suggest that they form a regulatory module (indicated by the dotted circle). (3) *Tc-foxQ2* and *Tc-six3* are both antagonized by Wnt/β-catenin signaling. (4) *Tc-six3* acts on Notch signaling via *Tc-ser*. (5/6) Mutual repression of *Tc-six3* and ocular *Tc-wg* expression. (7) Notch signaling-dependent activation of *Tc-foxQ2* is restricted to lateral parts of the anterior *Tc-foxQ2* expression. (8) Regulatory activities of *Tc-foxQ2* and *Tc-six3* are similar with respect to several downstream targets. (9) *Tc-foxQ2* but not *Tc-six3* is repressed by *Tc-croc* activity. (10) An unknown factor ‘X’ is predicted to activate the posterior part of the *Tc-croc* expression, while *Tc-six3* and *Tc-foxQ2* are required for the anterior portion. (11) *Tc-rx* is repressed by *Tc-foxQ2* but is not regulated by *Tc-six3*. Note that the assumed repressive effect is based on non-overlapping expression of these genes and the effects found in misexpression experiments (see text for conflicting RNAi data). (12) The late effect of *Tc-foxQ2* on *Tc-scro*, observed in gain-of-function experiments, is most likely secondary and is, hence, not considered here. (13) Notch signaling is involved in labrum development by regulating cell proliferation.

With this work we present the first functional analysis of *foxQ2* in protostomes and, together with previous work, we present the most comprehensive aGRN in that clade (Fig. 10). We found that *Tc-foxQ2* is required at the top of the gene regulatory network to pattern the anteriormost part of the beetle embryo.

Interestingly, we identified a positive regulatory loop between *Tc-six3* and *Tc-foxQ2* at early embryonic stages (germ rudiment), forming a novel regulatory module. In support of this, several genetic interactions with downstream genes are similar (Posnien et al., 2011b) (see Fig. 10). The *six3/foxQ2* regulatory module regulates a large number of genes that are required for anterior AMR patterning (e.g. anterior *Tc-cnc* and *Tc-croc*) and neuroectoderm patterning (e.g. *Tc-chx*). Hence, it lies at the top of the aGRN governing insect anterior head and brain development and both components elicit similar phenotypes when knocked down. Such a self-regulatory module at the top of a gene network is not without precedence. For instance, *eyes absent*, *eyeless*, *dachshund* and *sine oculis* form a positive regulatory loop at the top of the *Drosophila* eye gene regulatory network and, in consequence, all four genes are required for eye development and have similar mutant phenotypes (i.e. complete loss of the eyes) (Wagner, 2007). With our data, we are not able to distinguish between alternative modes of interaction: *Tc-foxQ2* and *Tc-six3* could cooperate in the regulation of the same targets, they could individually regulate different subsets, or one gene could be the regulator of all target genes while the other would just be required for the initiation of expression.

Within the regulatory module, *Tc-six3* appears to be the more upstream of the two based on a number of criteria. First, *Tc-six3* expression starts a bit earlier than that of *Tc-foxQ2* (at the differentiated blastoderm stage, as compared with the germ rudiment for *Tc-foxQ2*), and it has a larger expression domain, within which early *Tc-foxQ2* is completely nested. Further, the loss of *Tc-foxQ2* in *Tc-six3* RNAi is more complete, even at later stages, than the loss of *Tc-six3* in *Tc-foxQ2* in RNAi (Fig. 6). Finally, the *Tc-six3* cuticle phenotype is more penetrant and comprises a slightly larger region of the dorsal head (compare with Posnien et al., 2011b).

Interestingly, the mutual activation of the *six3/foxQ2* module does not persist, as the initially overlapping expression patterns diverge to become largely distinct at later embryonic stages (Fig. 5Bc-g). Just small domains in the neuroectoderm and the labrum continue to co-express both genes (encircled in Fig. 5Bd-g). Given their almost mutually exclusive expression, they might even switch to mutual repression at these later stages. In line with this scenario, the heat shock-induced misexpression of *Tc-foxQ2* led to a reduction in lateral aspects of *Tc-six3* expression (Fig. S15). Hence, it could be that later repression of *six3* by *foxQ2* is conserved between sea urchin and beetles.

### Protostome *foxQ2* has evolved novel functions in head and brain development

Functional studies of *foxQ2* orthologs were restricted to a sea urchin, as a model for deuterostomes, and the sea anemone, a cnidarian, representing the sister group to the bilaterian animals. This functional study in protostomes allows the first conclusions to be drawn on the evolution of *foxQ2* function. Compared with sea urchin and sea anemone, *Tc-foxQ2* plays a much more important role in the aGRN of our protostome model. First, it is clearly required for epidermal development, as evidenced by the loss of the entire labrum in knockdown animals. This is in contrast to sea urchin and sea anemone, where no epidermal phenotype was described apart from a thickened animal plate (Sinigaglia et al., 2013; Yaguchi et al., 2008). Second, *Tc-foxQ2* is required for the development of two brain parts involved in higher-order processing, namely the CX and the MBs. In other models, *FoxQ2* affects specification of certain neural cell types but does not lead to tissue loss. (Sinigaglia et al., 2013; Yaguchi et al., 2008, 2010). Finally, *Tc-foxQ2* is required for *Tc-six3* expression in *Tribolium*, which is not the case in the other models (Range and Wei, 2016; Sinigaglia et al., 2013; Yaguchi et al., 2008). Many of these novel functions may be explained by *Tc-foxQ2* gaining control over *Tc-six3* expression in our protostome model system.

### The evolutionary scenario: gain of *foxQ2* functions in animal evolution

Based on previous expression data, it has been suggested that *foxQ2* orthologs played a role in the anterior development of all animals and that this involved interaction with *six3* orthologs (Fig. S16) (Fritzenwanker et al., 2014; Hunnekuhl and Akam, 2014; Marlow et al., 2014; Martín-Durán et al., 2015; Santagata et al., 2012; Sinigaglia et al., 2013; Tu et al., 2006; Wei et al., 2009). In line with a conserved function, at early stages *foxQ2* shows co-expression with *six3* in deuterostomes and cnidarians and a nested expression within protostomes. In all cases, *foxQ2* arises within the *six3* domain (Fig. S16, left column). At later stages, by contrast, expression is more diverse. Some species retain nested or co-expression with *six3* (Sinigaglia et al., 2013; Marlow et al., 2014; Martín-Durán et al., 2015), whereas other species develop *six3*-negative/*foxQ2*-positive domains (Hunnekuhl and Akam, 2014; Martín-Durán et al., 2015; Fritzenwanker et al., 2014; Wei et al., 2009; Yaguchi et al., 2008), similar to what we found in the beetle. In other species, the anteriormost region is cleared of expression of both genes (Fig. S16, right columns). This variation is not clearly linked to certain clades, indicating that the regulatory interactions at later stages might have evolved independently. In addition to the novel functions described above, there are conserved functional aspects as well. Initial activation of *foxQ2* by *six3* is found in all three model species. Repression of *six3* by *foxQ2* was found in *Strongylocentrotus* and *Tribolium* (at later stages) but not *Nematostella* (Range and Wei, 2016; Sinigaglia et al., 2013).

Together, these data indicate that at the base of metazoans *foxQ2* and *six3* were involved in early anterior patterning, with *six3* being upstream of *foxQ2*. In bilaterians, the expression of both genes might have become restricted to the anterior by repression by posterior Wnt signaling (Leclère et al., 2016; Darras et al., 2011; Fritzenwanker et al., 2014; Marlow et al., 2014; Range and Wei, 2016; Sinigaglia et al., 2013; Wei et al., 2009; Yaguchi et al., 2008). After the split from Cnidaria, the Urbilateria *foxQ2* evolved a repressive function on *six3*, leading to more complex expression and increased diversity of the molecular code specifying cells at the anterior pole (Range and Wei, 2016). This diversification might have been required for the evolution of more diverse neural cell types. In protostomes, *foxQ2* additionally evolved control over early *six3* expression. Indeed, that *six3* is slightly more upstream of *foxQ2* in the regulatory module of *Tribolium* might be a remnant of its ancestrally more important role. A curiosity is the loss of such a highly conserved gene in the genome of placental mammals, whereas other vertebrates still have the gene (Mazet et al., 2003; Yu et al., 2008). Unfortunately, *foxQ2* function in vertebrates remains unstudied and our evolutionary hypothesis needs to be tested in other models representing deuterostomes, Lophotrochozoa and Ecdysozoa.

## MATERIALS AND METHODS

### Animals

Animals (*Tribolium castaneum*) were reared under standard conditions at 32°C (Brown et al., 2009). The *San Bernadino* (*SB*) wild-type strain was used for all experiments, except for initial reproduction of the phenotype, where the *black* (Sokoloff, 1974) and the *Pig-19/pBA19* (Lorenzen et al., 2003) strains were used as in the iBeetle screen. The Tc-*vermillion^white^* (*v_w_*) strain (Lorenzen et al., 2002) was used for transgenesis and heat shock experiments. Transgenic lines *MB-green* line (G11410) and *brainy* marking parts of the brain were described previously (Koniszewski et al., 2016).

### Sequence and phylogenetic analysis

*Tc-foxQ2* full coding sequence (1633 bp; GenBank accession number XM_008202469) was obtained from the *Tribolium* genome browser (http://bioinf.uni-greifswald.de/gb2/gbrowse/tcas5/) and the sequence was confirmed by cloning the full coding sequence from cDNA. Phylogenetic analysis was performed using MEGA v.5 (Tamura et al., 2011). The multiple sequence alignment was conducted with the ClustalW algorithm using the parameters preset in MEGA v.5. Positions containing gaps were eliminated from the dataset. The phylogenetic tree was constructed using the neighbor-joining method with the Dayhoff matrix-based substitution model (Schwartz and Dayhoff, 1979). Bootstrap tests (Felsenstein, 1985) were conducted using 1000 replicates to test the robustness of the phylogenetic tree. *Tc-Lin31* was the second best hit in the NCBI BLASTp (Altschul et al., 1997) search (http://blast.ncbi.nlm.nih.gov/Blast.cgi) and used as outgroup.

### RNAi

To test for RNAi efficiency, we examined *Tc-foxQ2* mRNA in *Tc-foxQ2* RNAi embryos (6-26 h AEL) by *in situ* hybridization (ISH). As expected, no signal was detected using regular detection settings (not shown) but increased exposure time revealed residual *Tc-foxQ2* expression at advanced embryonic stages (Fig. S5, note the increased background in B,D,F,H). These domains reflected only part of the WT expression pattern, indicating autoregulatory interactions restricted to some domains. Our subsequent analyses focused on early patterning, where the RNAi knockdown was shown to be very efficient (Fig. S5A,B). Furthermore, RT-qPCR (supplementary Materials and Methods) experiments confirmed efficient knockdown of the *Tc-foxQ2* mRNA level (−91.3%) using the *Tc-foxQ2*^RNAi_a^ dsRNA fragment (Fig. S6). Embryonic RNAi phenotypes were found with a penetrance of over 90%, except for *Tc-foxQ2*, *Tc-rx* and *Tc-six4*, which showed a penetrance of over 40%.

The templates for the non-overlapping dsRNA fragments used in this study were generated by PCR from a plasmid template (for primer and dsRNA template sequences see Table S8). The dsRNAs were synthesized using the MEGAscript T7 Transcription Kit (Invitrogen) or purchased from Eupheria BioTech. The transcribed dsRNA was extracted via isopropanol precipitation (*Tc-foxQ2*^RNAi_a^) or phenol/chloroform extraction (*Tc-foxQ2*^RNAi_b^) and dissolved in injection buffer (1.4 mM NaCl, 0.07 mM Na_2_HPO_4_, 0.03 mM KH_2_PO_4_, 4 mM KCl, pH 6.8). The injected dsRNA concentrations for parental RNAi with *Tc-foxQ2*^RNAi_a^ and *Tc-foxQ2*^RNAi_b^ were 1.0 µg/µl, 1.5 µg/µl and 3.1 µg/µl. Unless stated otherwise, 1.5 µg/µl dsRNA was used. Pupal injections were performed as previously described (Bucher et al., 2002; Posnien et al., 2009b). The dsRNA was injected using FemtoJet Express (Eppendorf). Cuticles of the L1 larval offspring were prepared as described (Wohlfrom et al., 2006).

### Staining

Standard immunostaining was performed using the cleaved *Drosophila* Dcp-1 (Asp216) rabbit antibody (Cell Signaling Technology, #9578) at 1:100 dilution. Anti-rabbit secondary antibody coupled with Alexa Fluor 488 (Thermo Fisher Scientific, A-11070) was used for detection at 1:1000 dilution. ISH (alkaline phosphatase/NBT/BCIP) and double ISH (alkaline phosphatase/NBT/BCIP, and horseradish peroxidase-mediated TSA reaction; the Dylight550 conjugate was synthesized by Georg Oberhofer) were performed as described previously (Oberhofer et al., 2014; Schinko et al., 2009; Siemanowski et al., 2015).

### Quantification of apoptosis

The regions of interest were set based on head morphology. Cell counting was performed using the Fiji cell counter plug-in (Schindelin et al., 2012). The number of apoptotic cells was positively correlated with age. Hence, to circumvent systematic errors due to staging, the apoptotic cell number in the posterior procephalon was used to normalize the data. This region was chosen because it was outside the *foxQ2* expression domain and unaffected by our RNAi experiments. The correction value was calculated by dividing the mean number of apoptotic cells of RNAi embryos by the mean number of apoptotic cells in WT embryos in the control region. For the normalization, the data were divided by the correction value. Raw counts are shown in Table S7.

The normalized data were tested with R (v.2.14.2; http://www.R-project.org/) for the homogeneity of the variances via the box plot, and for normal distribution via the Shapiro-Wilk test. To test for significance, three statistical tests were conducted: Welch's *t*-test, two-sample *t*-test, and the Wilcoxon rank-sum test. All three tests showed the same levels of significance. *P*-values are based on the Wilcoxon rank-sum test results.

### Transgenesis and heat shock

The *foxQ2* heat shock construct was based on the constructs developed by Schinko et al. (2012) and germline transformation was performed as described previously (Berghammer et al., 1999; Schinko et al., 2012) using the injection buffer used for RNAi experiments and the mammalian codon-optimized hyperactive transposase (Yusa et al., 2011) flanked by the *Tc-hsp68* sequences (gift from Stefan Dippel, Göttingen University). All animals for heat shock experiments were kept at 32°C. Heat shock was performed as described previously (Schinko et al., 2012) for 10 min at 48°C, with egg collections for cuticle preparations at 0-24, 9-13, 14-20 and 20-25 h AEL, and egg collections for ISHs at 9-13 h AEL (fixed 5 h after heat shock). For each gene, one batch of embryos was used. Phenotypes shown in figures were exhibited by over 50% of the embryos in the batch.

### Image documentation and processing

Cuticle preparations and L1 larval brains were imaged as described (Posnien et al., 2011b; Wohlfrom et al., 2006) using an LSM 510 or Axioplan 2 microscope (Zeiss) and processed using Amira (v.5.3.2; FEI) using ‘voltex’ projections. Stacks were visualized as average or maximum projections using Fiji (v.1.49i). All images were level-adjusted and assembled in Photoshop CS (Adobe) and labeled using Illustrator CS5 (Adobe).
